# Development and Validation of a Novel Forensic STR Multiplex Assay for Domestic Pigeon (*Columba livia domestica*)

**DOI:** 10.3390/ijms27188324

**Published:** 2026-09-19

**Authors:** Qian Zhou, Weiheng Xiao, Man Chen, Lei Jiang, Weifen Sun, Yifei Zeng, Jiamei Jiang, Xiangping Li, Liping Hu, Xiling Liu

**Affiliations:** 1 Department of Forensic Genetics, College of Forensic Medicine, Kunming Medical University, Kunming 650500, China; 2 Shanghai Key Laboratory of Forensic Medicine, Shanghai Forensic Service Platform, Academy of Forensic Science, Shanghai 200063, China

**Keywords:** STR, domestic pigeon, individual identification

## Abstract

The domestic pigeon (*Columba livia domestica*), recognized as one of the earliest species to be domesticated by humans, has sustained a profound and enduring association with human society. These birds fulfill significant social and economic roles, particularly in the context of pigeon racing competitions. To ensure fairness and authenticity in such events, as well as to address concerns related to meat fraud and adulteration, we have developed a six-color fluorescent multiplex PCR amplification system incorporating 24 short tandem repeat (STR) loci and the chromobox helicase DNA-binding gene (CHD) specific to domestic pigeons. The multiplex assay underwent comprehensive validation in accordance with the guidelines established by the Scientific Working Group on DNA Analysis Methods (SWGDAM) and the Committee on Wildlife Forensic Science Guidelines, encompassing evaluations of PCR conditions, precision, species specificity, sensitivity, stability, repeatability, and reproducibility. Furthermore, population genetic analyses and evaluations using simulated forensic case samples were conducted. The assay’s applicability to closely related species was also evaluated. Our results show that the developed pigeon STR genotyping system exhibits high levels of accuracy, specificity, repeatability, stability, and robustness. Complete genotyping profiles were obtained with only 125 pg of domestic pigeon genomic DNA. Analysis of 248 unrelated domestic pigeons obtained from a local market in Shanghai, China, yielded the cumulative probability of exclusion (CPE) and the combined power of discrimination (CPD) values of 0.999999659383865638452 and 0.99999999999999999999918448912103896, respectively. Additionally, the majority of primers effectively amplified loci in congeneric species, indicating the assay’s broad applicability. These results suggest that the multiplex system is highly polymorphic and well-suited for individual identification and kinship analysis in domestic pigeons, with potential applicability extending to related species.

## 1. Introduction

The domestic pigeon, *Columba livia domestica*, constitutes the most extensively distributed avian species globally and is widely recognized as a symbol of peace [1]. It is among the earliest bird species to have been domesticated by humans, maintaining a longstanding association with human societies and inhabiting both urban and rural environments extensively [1,2]. This domesticated form originates from the rock pigeon (*Columba livia*), a species within the genus Columba of the family Columbidae, which is native to regions spanning Europe, the Middle East, South Asia, and North Africa [1]. Genomic and archaeological evidence indicates that rock pigeons were initially domesticated in the Middle East primarily for food purposes and exhibit genetic affinities with Middle Eastern rock pigeon populations [1,3,4,5,6]. Over millennia of selective breeding, hundreds of domestic pigeon breeds have emerged worldwide, rendering them among the most phenotypically diverse domesticated avian species [1,7].

Domestic pigeons fulfill important social roles and possess considerable economic significance [8]. They are generally classified into three categories based on their primary utility: meat pigeons, fancy pigeons, and racing pigeons. Meat pigeons are bred for consumption and are further subdivided by body weight into standard meat pigeons (typically weighing 400–500 g, exemplified by the Silver King breed) and giant meat pigeons (adults often exceeding 700 g, such as the Salonta Giant) [9]. Fancy pigeons, characterized by their ornamental features, include breeds such as the Jacobin [1]. Racing pigeons, also referred to as homing pigeons, exhibit exceptional homing abilities and directional orientation, capable of covering distances exceeding 1000 km daily at speeds surpassing 100 km/h [10]. Historically employed for message delivery during wartime, these birds are currently primarily engaged in competitive racing. In addition, feral pigeon populations, derived from escaped or abandoned domestic pigeons, have established themselves in urban areas [11]. Despite pronounced phenotypic variation among domestic pigeon breeds in traits such as vocalizations, body size, plumage, and coloration, genetic analyses reveal limited differentiation between breeds and evidence of genetic admixture [12,13]. Moreover, domestic pigeons serve as valuable experimental models in biological and behavioral studies [3].

Pigeon racing represents a longstanding and widespread tradition [14]. With its expansion, associated economic activities, including auctions and breeding, have also increased. However, the substantial financial stakes involved have led to frequent instances of fraud, such as substituting inferior birds for superior ones, thereby undermining the fairness of competitions and adversely affecting participants’ economic interests [15]. To preserve the integrity and authenticity of pigeon racing events, reliable methods for individual pigeon identification are essential. Various pigeon racing associations worldwide have implemented the use of unique computer-coded leg bands for each racing pigeon to mitigate misidentification. Although leg band numbers facilitate straightforward individual identification under normal conditions, they remain susceptible to forgery, replacement, and tampering, thus failing to provide unequivocal evidence. Concurrently, rising consumption levels have driven steady growth in demand for pigeon meat, accompanied by increased emphasis on meat quality and authenticity. Nonetheless, incidents of meat fraud and adulteration persist, exemplified by Europe’s “horse meat scandal” [16]. Consequently, the development of accurate and reliable pigeon identification technologies is crucial for preventing such malpractices, safeguarding competition fairness, and ensuring meat authenticity.

Currently, DNA molecular markers employed in domestic pigeon genetics and forensic investigations include the cytochrome oxidase subunit I gene (COI) and short tandem repeats (STR). The COI gene is widely regarded as a highly reliable genetic marker due to its extensive application in vertebrate species identification, particularly for distinguishing among species [17,18]. However, COI is suboptimal for differentiating domestic pigeon populations owing to its high sequence conservation across different groups and low genetic differentiation within the species [19]. STRs, also known as microsatellite loci, have proven to be efficient and precise tools for individual identification and parentage verification, applicable in routine testing and legal contexts [20,21]. STR loci represent the preferred genetic markers across various animal species [22,23]. In domestic pigeons, STRs are extensively used to investigate genetic structure and diversity and constitute the principal markers for individual identification and parentage analysis [5,12,24,25,26]. The International Society for Animal Genetics (ISAG), an organization dedicated to advancing animal genetics research and facilitating knowledge exchange, recommended in 2014 a panel of 12 core loci and 4 supplementary loci for individual identification in domestic pigeons. These 16 loci remain the standard for forensic identification in this species, having been validated by laboratories internationally and demonstrating high practicability. Research on STR loci in domestic pigeons dates back to 2000, when Traxler et al. [27] employed seven microsatellite loci prefixed with “Cliμ” to elucidate the breeding origins of the Vienna Highflyer and to assess genetic relationships among strains. In 2007, Lee et al. [15] developed a composite STR system comprising seven microsatellite loci prefixed “PG” alongside a CHD (chromodomain helicase DNA-binding gene) marker, showing promise for individual identification and kinship analysis in domestic pigeons. Ramadan et al. [26] further explored the genetic diversity of Egyptian pigeons based on these STR loci. Subsequent studies have identified additional loci suitable for domestic pigeon research [13,28,29]. For instance, Zhang et al. [28] developed microsatellite marker systems for multiple avian species, including six STR loci specific to domestic pigeons.

In this study, a comprehensive review of relevant literature and databases was conducted to compile known STR loci in domestic pigeons. We incorporated the pigeon sex-determining locus (chromodomain helicase DNA-binding gene, CHD) and selected loci appropriate for forensic identification of pigeon populations. A novel multiplex STR genotyping amplification system was developed, aiming to provide an innovative tool for forensic non-human identification in pigeons.

## 2. Results

### 2.1. Characterization of STR Markers in Domestic Pigeons

**Figure 1:**
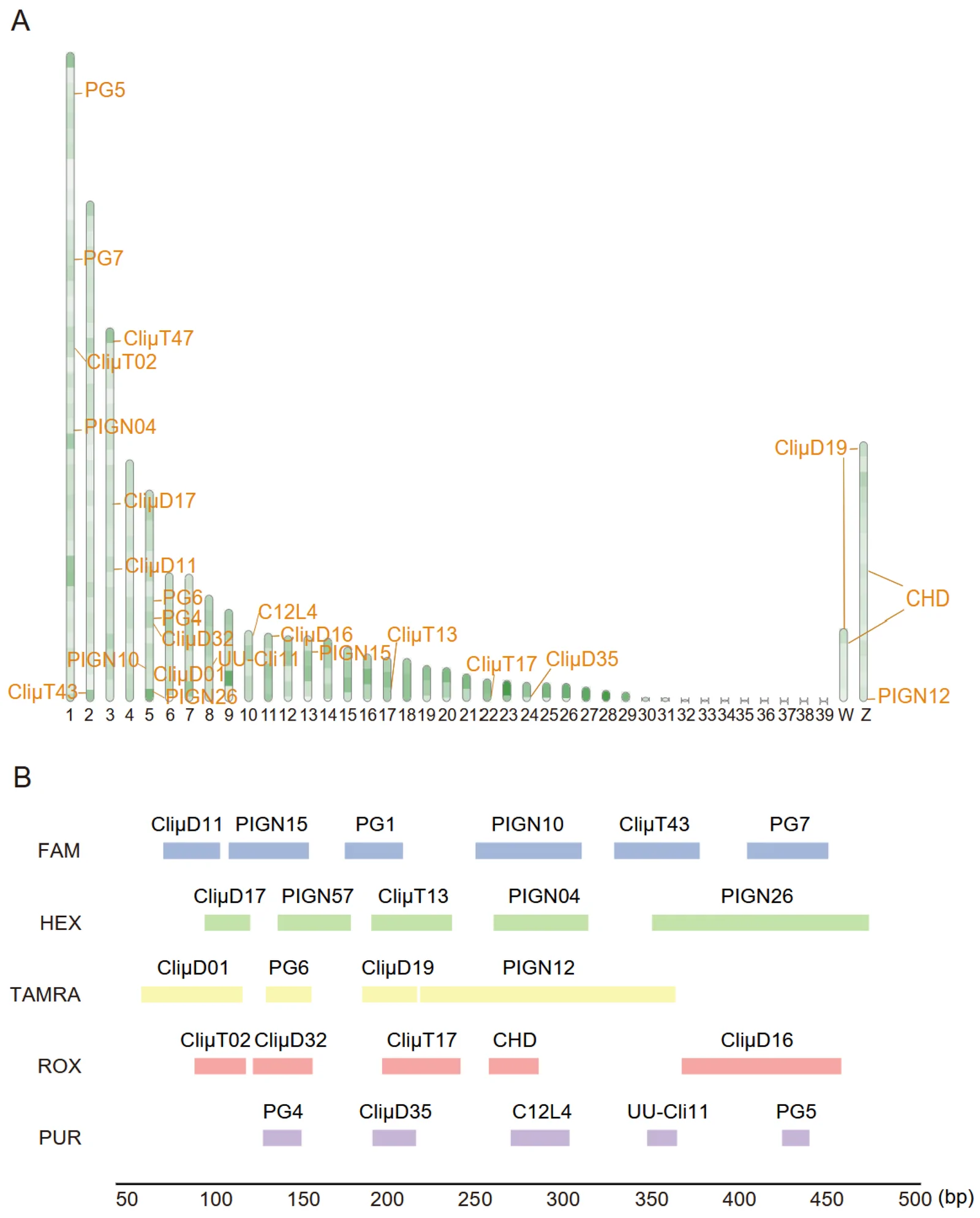
Characteristics of the developed system: (**A**) The chromosomal positions of the 24 STR loci and the CHD locus. (**B**) A schematic diagram of the marker arrangement and dye labeling scheme for the 24 STR loci and the CHD locus.

A total of 24 STR loci, namely CliµD35, PG5, UU-Cli11, PG6, CliµT02, CliµD19, CliµD16, CliµD17, PG1, PG4, CliµD11, PG7, C12L4, CliµT43, PIGN57, CliµT17, CliµT13, PIGN04, CliµD01, CliµD32, PIGN15, PIGN12, PIGN10 and PIGN26, alongside the CHD locus, were selected from domestic pigeon samples. Except for the PIGN57 locus, these STR loci are distributed across 13 distinct chromosomes (Figure 1). Among these loci, seven contain dinucleotide repeats, two possess trinucleotide repeats, twelve exhibit tetranucleotide repeats, and one features a pentanucleotide repeat motif (Figure S1). Notably, there are two loci (PIGN10 and PIGN12) with compound STR motifs characterized by the nucleotide repeat patterns (TC)_n_(TA)_n_ and (TC)_n_(TC)_n_(TA)_n_, respectively. Comprehensive details regarding primer sequences, chromosomal localization, and repeat motifs for each STR and the CHD loci are provided in Table S1.

### 2.2. Development of a Multiplex PCR System

A novel genotyping system was developed for domestic pigeons to enable individual-level sex differentiation. The constructed allelic ladder is shown in Figure S2. Valid genotyping data were obtained for all 248 domestic pigeon DNA samples. The genotype profile of individual CL-28, shown in Figure S3, indicates a female genotype (CHD: ZW). Among the loci examined, sixteen (CliμD11, PIGN15, PG1, PIGN10, CliμT43, PG7, CliμD17, PIGN57, CliμT13, PIGN04, PIGN26, CliμD01, CliμD32, CliμT17, PG4, and C12L4) were heterozygous, whereas eight loci (PG6, CliμD19, PIGN12, CliμT02, CliμD16, CliμD35, UU-Cli11, and PG5) were homozygous.

### 2.3. Species-Specificity Assessment

To evaluate the specificity of the STR system and its ability to prevent genotyping interference from non-target species, eleven common species were tested following standardized procedures. No specific amplification products were detected in nine of these species (Figures S4 and S5). However, two species exhibited non-specific amplification products across three biological replicates each. Specifically, off-ladder (OL) peaks were observed at the CHD locus in chicken samples, while OL peaks appeared at both the CHD and PG4 loci in duck samples (Figure S4). Among the additional five avian species, *Spilopelia chinensis* exhibited typing results at all loci except CliµD11, CliµD17, PIGN4, PIGN26, CliµD01, CliµD19, PIGN12, CliµD32, CliµT17, and CliµD, and its profiles partially overlapped with those of *Columba livia domestica*. This overlap may be due to their close phylogenetic relationship, implying a relatively close genetic affinity. In contrast, the other four species, despite occasionally producing single peaks at individual loci (e.g., CHD), displayed profiles that diverged substantially from the domestic pigeon, with alleles positioned far outside the domestic pigeon bin ranges, thereby facilitating unambiguous discrimination (Figure S5).

### 2.4. Sensitivity Evaluation

**Figure 2:**
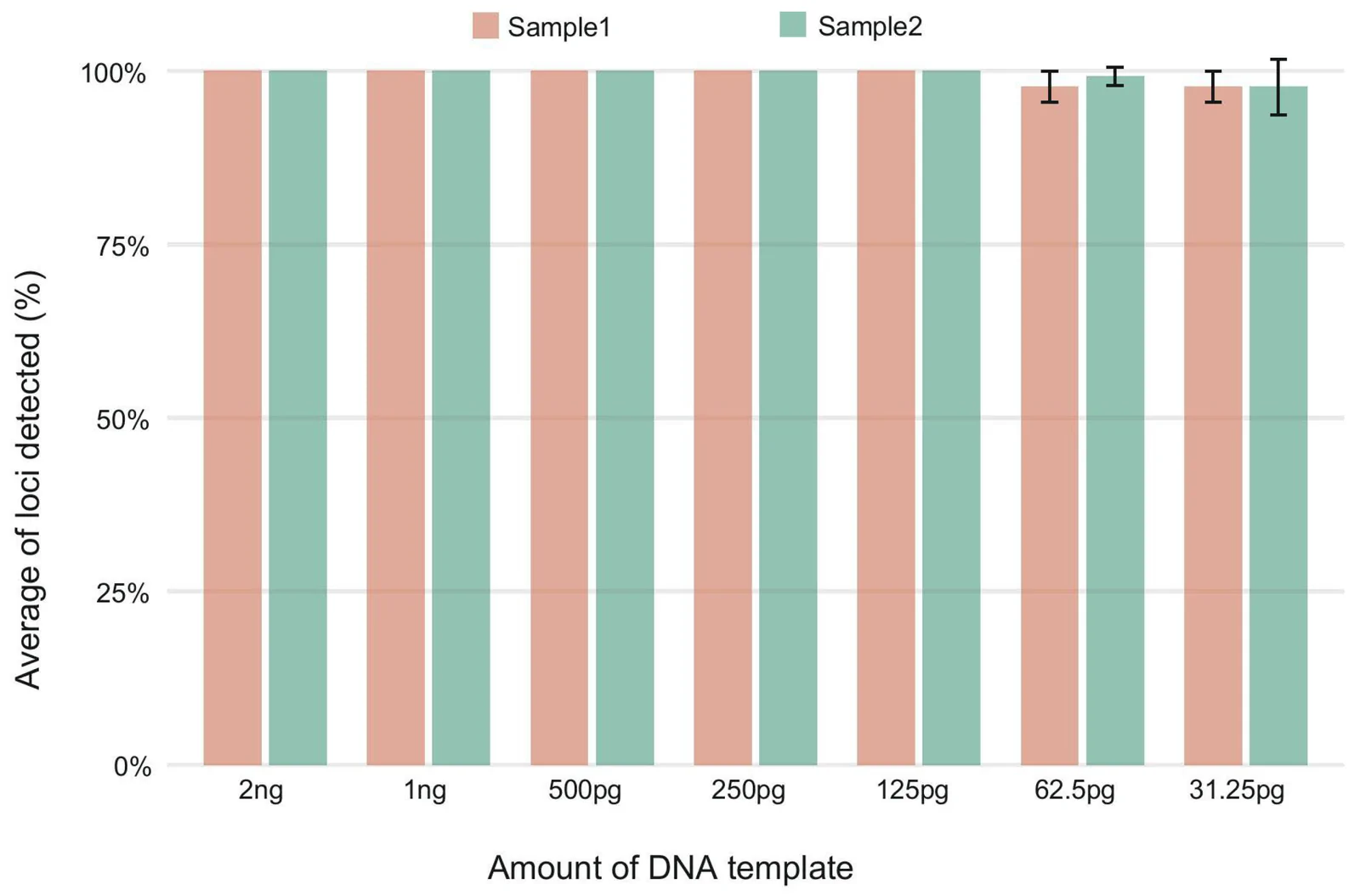
Bar graph of sensitivity assessment. This test evaluated the average allele detection rates across seven serial dilutions of sample DNA, ranging from 2 ng to 31.25 pg. Error bars indicate the standard deviation of three replicates. Orange represents Sample 1, and green represents Sample 2.

The sensitivity of the system was assessed using two domestic pigeon DNA samples with input quantities ranging from 2 ng to 31.25 pg. Complete genotype profiles were consistently obtained at DNA inputs ≥125 pg (Figure 2). At 62.5 pg, allele dropout was observed, with average allele detection rates of 97.72% and 97.67% for the two samples, respectively. In the DNA degradation assay, allelic dropout was first detected in the 5-min treatment group. The number of loci showing dropout increased progressively as irradiation time was extended. After 60 min of UV exposure, individual 1 showed complete allelic dropout, whereas individual 2 retained only three loci (Table S2).

### 2.5. Precision Analysis

**Figure 3:**
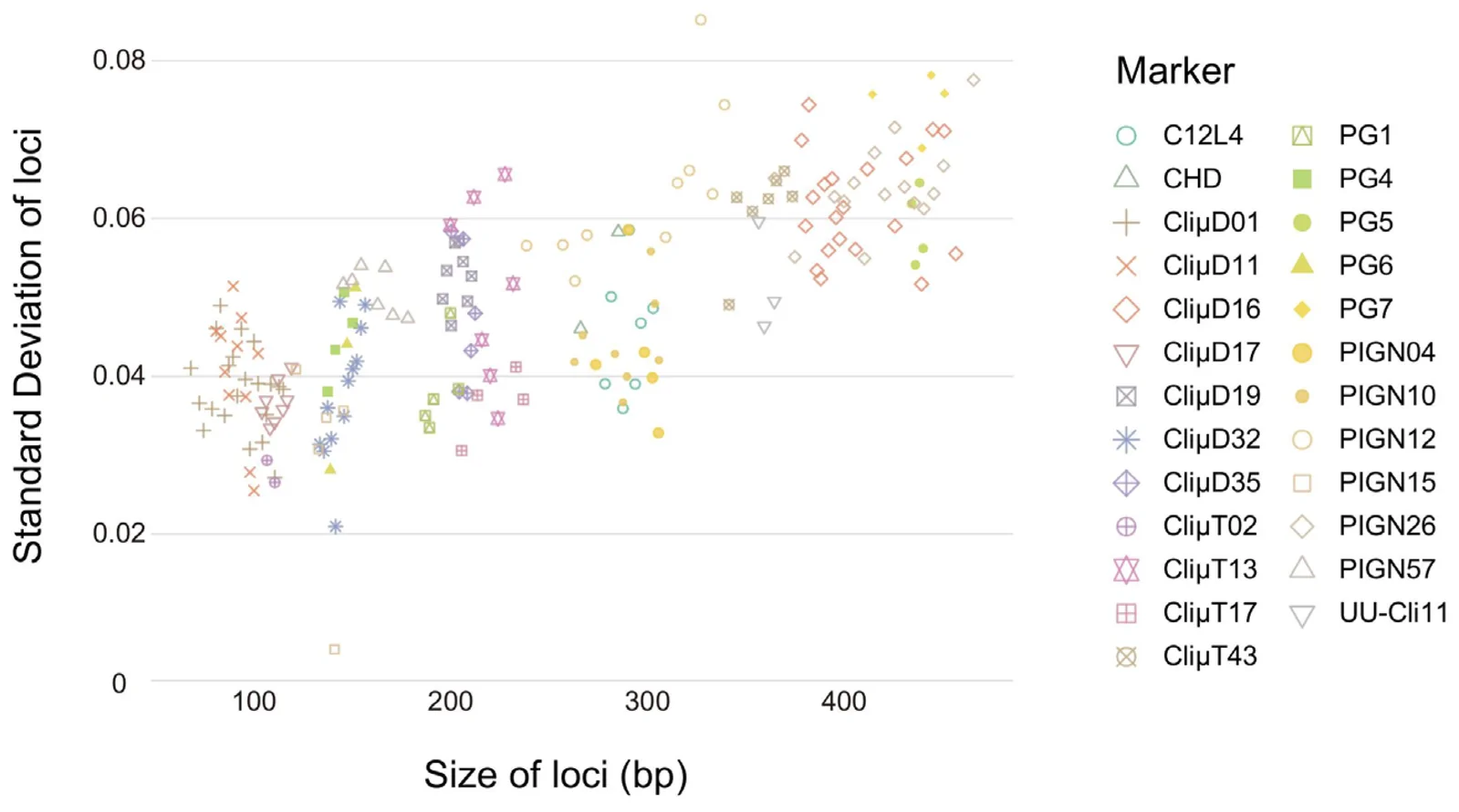
Size precision evaluation. The x-axis represents the allele size, and the y-axis represents the standard deviation of each allele’s size measurement.

The precision of the allelic ladder was evaluated using the ABI 3500xL Genetic Analyzer. The standard deviation of allele sizing ranged from 0.0054 (PIGN15) to 0.0852 (PIGN12), with the overall SD not exceeding 0.0776 and 3 × SD not exceeding 0.2328 (Figure 3 and Table S3).

### 2.6. Stability Testing

**Figure 4:**
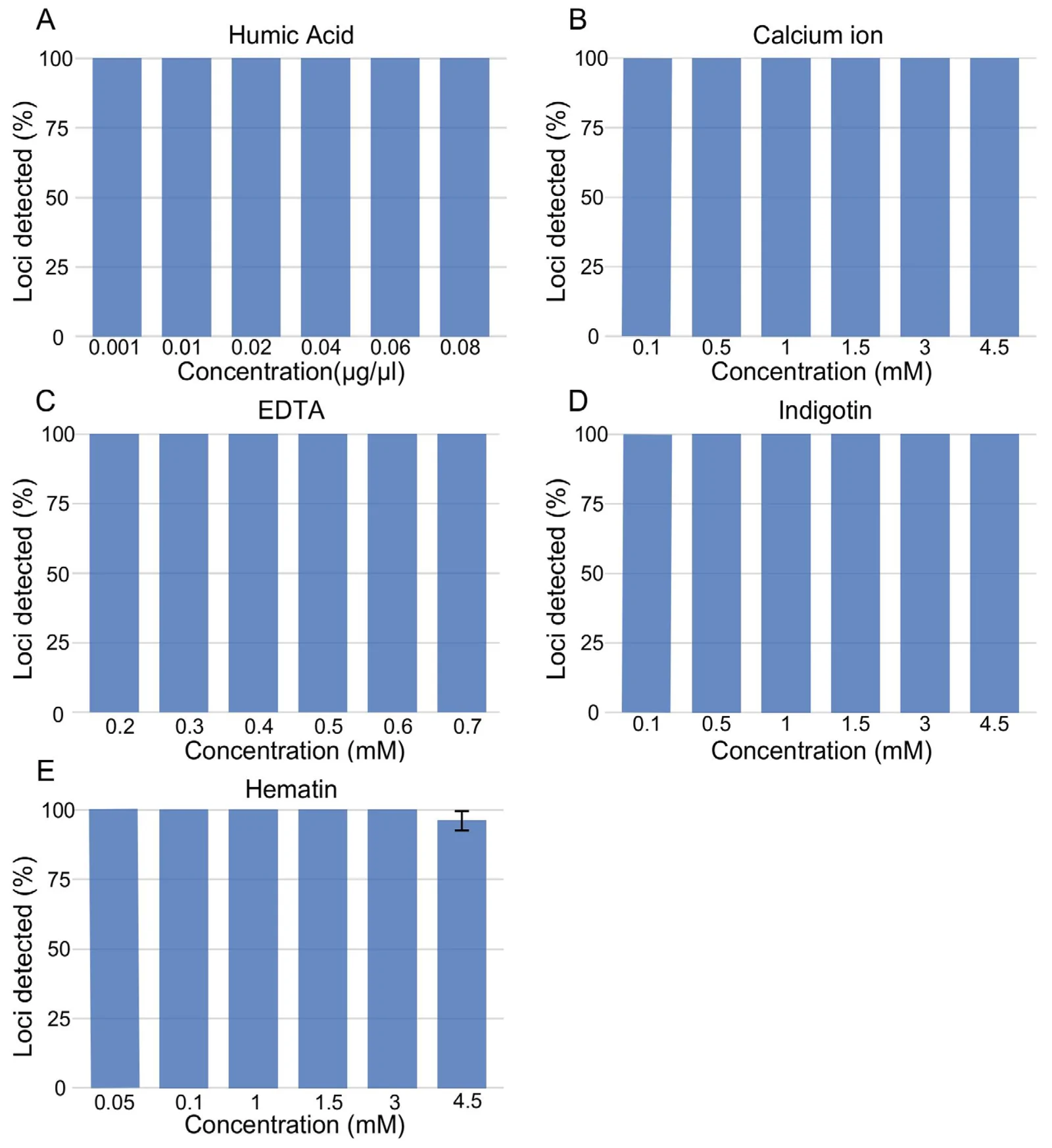
Stability assessment. This system’s allele detection rates were measured in sample DNA subjected to varying concentrations of five different inhibitors: (**A**) humic acid; (**B**) calcium ion; (**C**) EDTA; (**D**) indigotin; (**E**) hematin. The x-axis represents the concentration gradients for each inhibitor, whereas the y-axis shows the mean allele detection rate at each concentration. Error bars indicate the positive and negative standard deviations from three replicate experiments.

The robustness of the STR system against common PCR inhibitors was examined by introducing six inhibitors (Hematin, Calcium ion, Humic Acid, Indigotin, and EDTA) into DNA samples at an input of 0.5 ng (Figure 4). Calcium ions, humic acid, indigotin, and EDTA did not adversely affect genotyping, maintaining average allele detection rates of 100% across all tested concentrations. Hematin showed no detrimental effect up to 3 mM. However, a decline in detection rate was observed at 4.5 mM. It should be noted, however, that the possible inhibitory effect of the low NaOH concentration in the solvent cannot be completely excluded.

### 2.7. Repeatability and Reproducibility

Repeatability and reproducibility were assessed by two independent operators who performed technical triplicates on domestic pigeon DNA samples under identical experimental conditions as described in Section 2.5 and Section 2.6. All replicates yielded complete and consistent STR genotyping results.

### 2.8. Concordance Analysis

Genotyping of DNA samples derived from various organs of domestic pigeons, including blood, liver, myocardium, stomach tissue, skeletal muscle, and saliva, using this STR system produced complete and consistent results (Table S4).

### 2.9. Stutter Peak Analysis

Capillary electrophoresis data obtained from 248 domestic pigeon DNA samples were analyzed to characterize stutter peaks. Mean stutter peak ratios and their corresponding standard deviations were calculated for both minus and plus stutter peaks across the 24 STR loci (Table S5). The highest average peak height ratio was identified at the CliµD01 locus, measuring 37.46%. Eight loci, namely CliµD01, CliµD17, CliµD35, CliµD19, CliµD16, CliµD32, CliµD11, and PG6, showed minus stutter ratios exceeding 15%, with CliµD01, CliµD17, and CliµD35 demonstrating a 100% occurrence rate of the minus stutter. No minus stutter peaks were detected at the PG5 and UU-Cli11 loci. Regarding plus stutter, the PIGN10 locus showed the highest average peak height ratio at 22.45%, with three loci (PIGN10, PIGN04, and CliµT17) exceeding the 15% threshold. The highest incidence of plus stutter was observed at the CliµD17 locus. Plus stutter peaks were detected in only a single sample at the CliµD16, PIGN04, PG7, and PIGN12 loci, whereas no plus stutter peaks were observed at the UU-Cli11, PG5, PG6, and CliµT43 loci.

### 2.10. Population Genetics Analysis

**Figure 5:**
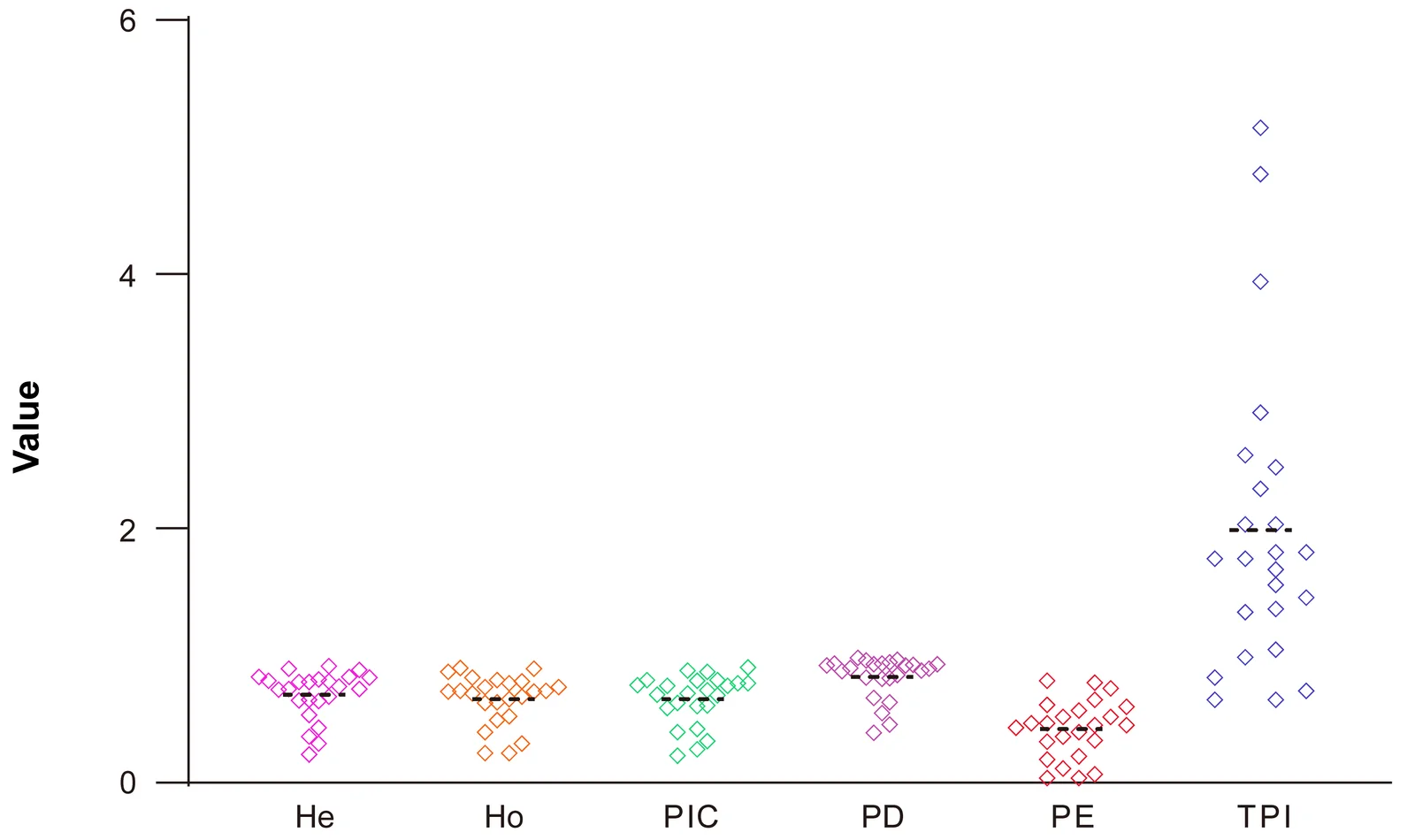
Forensic parameter distribution and interspecies identity analysis: the distribution of forensic parameters across 248 individual samples, including expected heterozygosity (He, pink); observed heterozygosity (Ho, orange); polymorphism information content (PIC, green); discrimination power (PD, purple); probability of exclusion (PE, red); Typical Paternity Index (TPI, blue). The x-axis lists the parameter names, and the y-axis represents their respective values. Dashed lines indicate the mean values for each parameter.

Population genetic parameters were evaluated for the 24 STR loci in a sample of 248 pigeons (Figure 5; Table S6). A total of 266 unique alleles were detected, with an average of 11.0833 alleles per locus, ranging from 2 alleles at locus PG5 to 34 alleles at locus PIGN12 (Table 1). Ho values spanned from 0.2661 to 0.9113, with a mean of 0.6594, while He values ranged from 0.2818 to 0.9115, averaging 0.6925. The mean polymorphism information content (PIC) was 0.6588, varying between 0.26 at locus CliµD35 and 0.9077 at locus PIGN26. Discrimination power (PD) values ranged from 0.4671 (PG5) to 0.9838 (PIGN26), with more than 79.17% of loci exhibiting PD values above 0.7. PE varied from 0.0505 (PG5) to 0.8185 (PIGN10), and TPI (Typical Paternity Index) ranged from 0.6813 (PG5) to 5.6364 (PIGN10). Following Bonferroni correction for multiple testing (*p* ≤ 0.05/276), 22 of the 24 loci conformed to Hardy–Weinberg equilibrium, whereas PIGN12 and CliµD19 were found to align with the sex chromosome region of the pigeon upon BLAST interrogation; these loci were omitted from the respective computations (Table S7). Linkage disequilibrium (LD) analysis revealed no significant associations after correction (Table S8). After removing PIGN12 and CliµD19, CPE and CPD values were 0.999999659383865638452 and 0.99999999999999999999918448912103896, respectively. Analysis of heterozygous peak height balance demonstrated average ratios ranging from 0.7113 to 0.9110 across loci (Table S9).

### 2.11. Cross-Species Applicability

To assess the cross-species applicability of the STR system within the genus Columba, sequence identity analyses were conducted for each STR locus, encompassing the core sequence as well as 500 bp of flanking regions both upstream and downstream. These analyses compared three Columba species with the domestic pigeon (*Columba livia domestica*). Primers targeting the CHD locus, originally designed for the domestic pigeon, failed to simulate amplification on the W chromosomes of *Columba palumbus* and *Columba guinea*. Similarly, primers for the PG5 locus did not yield simulated amplification products on the reference genome of *Columba guinea*. For loci where simulated amplification was successful, sequence identity values between the domestic pigeon and *Columba janthina*, *Columba palumbus*, and *Columba guinea* all exceeded 0.9 (Table S10). Additionally, examination of base repeat units across the reference genomes identified clear repeat motifs at all loci except for PIGN12 in *Columba janthina* and PG5 in *Columba palumbus,* where no distinct repeat units were observed (Table S11).

## 3. Discussion

In this study, we successfully developed an STR system for individual identification and kinship analysis in domestic pigeons. This system comprises 24 STR loci alongside the sex-determining CHD locus, facilitating comprehensive amplification of complete domestic pigeon genotypes. An allelic ladder was constructed, demonstrating allele size differences within 0.5 base pairs, thereby validating the system’s capacity for accurate and reliable allele detection. Genotyping across all 25 loci can be accomplished in a single PCR reaction with a total reaction time of approximately 1 h and 38 min. Evaluations of stability, repeatability, reproducibility, and identity confirmed the system’s robust and reliable performance. It shows strong tolerance to common PCR inhibitors and generated unique genotyping profiles for individual domestic pigeons, irrespective of sample type. Furthermore, the average heterozygote peak height ratio exceeded 70% at each locus, consistent with established criteria that consider ratios below 70% indicative of severe imbalance [30,31], thereby underscoring the balanced amplification efficiency of the system.

When including the PIGN12 and CliµD19 loci, these CPE and CPD values were 0.999999659383865638452 and 0.99999999999999999999918448912103896, respectively. Both calculation methods yielded exceptionally high values, indicating the system’s strong capability for pigeon identification and kinship analysis. In comparison, Wei et al. [32] developed a system comprising 23 STR loci with CPD and CPE values of 1 − 3.6664 × 10^−19^ and 1 − 6.5968 × 10^−5^, respectively. Our system exhibits superior CPE and CPD values relative to their system.

The inclusion of the sex-determining CHD locus was motivated by the fact that approximately 50% of avian species, including domestic pigeons, are monomorphic and cannot be sexed based on external morphology at juvenile or adult stages. To address the requirement for sex identification, we assessed the system’s sensitivity, establishing a detection limit of 125 pg. DNA input below this threshold in a 10 µL reaction volume may result in allele dropout or peak heights falling below the analysis threshold (set at 50 RFU). Species specificity was evaluated using DNA from 11 common species. Non-specific amplification was observed at the CHD locus in chickens and at both the CHD and PG4 loci in ducks, whereas no specific amplification was detected in the remaining nine common species, indicating the system’s robustness and low susceptibility to interference from non-target species DNA. Wei et al. similarly reported non-specific amplification at the CHD locus in chickens and ducks [32]; we speculate that this phenomenon may arise from the considerable sequence conservation in the flanking regions of the primer-binding sites in these avian taxa.

Taxonomically, domestic pigeons, chickens, and ducks belong to the class Aves, and CHD is conserved among non-ratite birds [33] with single-copy sequences located on the Z and W chromosomes. Electronic PCR (ePCR) analyses of chicken and duck reference genomes using primers designed for the domestic pigeon CHD locus confirmed amplification of similarly sized products. Although prior studies have not reported amplification at the PG4 locus in ducks, we hypothesize that unintended primer binding within the duck genome may explain this observation. ePCR analysis using pigeon PG4 primers on the duck genome revealed no amplification of similarly sized products, and optimal binding sites differed by five and six bases for the forward and reverse primers, respectively, effectively excluding the possibility that the anomalous band resulted from pigeon PG4 primer amplification. Subsequent ePCR with all other primers under identical fluorescent labeling conditions identified that the CliμD35 primers amplified products of similar size, with forward and reverse primer sequences differing by only three and zero bases, respectively, in the duck genome. Sequence analysis of the ePCR products revealed 94.9% identity between duck and pigeon regions, indicating that the CliμD35 primer binding sites are highly homologous and that flanking sequences at this locus are conserved between these species, accounting for the observed cross-species amplification.

To characterize the STR system within domestic pigeon populations, Hardy–Weinberg equilibrium (HWE) tests were performed. After Bonferroni correction (significance threshold set at *p* < 0.002), 22 autosomal STR loci conformed to HWE expectations (*p* > 0.002). Two loci, PIGN12 and CliµD19, were identified as sex-chromosome markers and therefore excluded from the above analysis.

Stutter peaks, which are common PCR artifacts observed during STR amplification, can complicate allele calling and thus necessitate careful analysis to ensure genotyping accuracy. The selected STR loci encompass repeat motifs ranging from dinucleotide to hexanucleotide units, with dinucleotide loci being particularly susceptible to stutter formation, potentially resulting in allele misinterpretation [34]. Accurate STR typing relies on the ratio of stutter peak height relative to the true allele peak height. Employing methodologies described in [35,36], we analyzed stutter data derived from 248 samples. In the analysis of minus stutter peaks, eight loci exhibited average stutter ratios exceeding 15%, with all but the PG6 locus showing high occurrence rates (>70%), indicating a pronounced susceptibility to elevated stutter product formation during PCR. Notably, all dinucleotide repeat loci were included among these eight, consistent with the known propensity of shorter repeat units to induce replication slippage [37]. The tetranucleotide peak locus PG6 also showed an average minus stutter ratio above 15%. However, minus stutter peaks were observed in only two samples, and no allelic miscalls were observed, suggesting that caution is warranted when interpreting alleles at this locus. Regarding plus stutter peaks, three loci showed mean stutter ratios exceeding 15%, although plus stutter peaks were detected in only one sample per locus, with no misinterpretations observed. Based on these observations, it is recommended that more stringent filtering criteria be applied to loci exhibiting high stutter occurrence and average peak height ratios above 15%. From the analysis of the 248 DNA samples, a reference for establishing stutter filtering parameters within this system was provided (Table S4).

The cross-species applicability of the STR system was further evaluated through bioinformatic approaches. Sequence identity analyses of each STR locus, including the core repeat and 500 bp flanking regions upstream and downstream, between domestic pigeons and other species within the genus Columba revealed identity values exceeding 0.9 for all loci amenable to amplification via ePCR, indicating evolutionary conservation and close phylogenetic relationships. These results suggest the potential for cross-species application of the STR system [38,39,40]. ePCR simulations predicted successful amplification of most STR loci across the reference genomes of three Columba species, supporting the system’s interspecies applicability. No amplification was detected at the CHD locus on the W chromosome of *Columba palumbus*, consistent with the fact that the reference individual was male (ZZ) and thus lacked a W chromosome, as expected under avian sex determination systems. Additionally, for the PG5 locus of *Columba palumbus* and the PIGN15 locus of *Columba janthina*, sequence alignment failed to identify homologous regions, possibly reflecting genomic evolutionary events such as gene loss or chromosomal rearrangements [27,40]. Similarly, *Columba guinea* showed no amplification at the W-chromosomal CHD locus despite being female (ZW). This absence may be attributed to (1) the contig-level assembly of the *Columba guinea* reference genome, which is fragmented and incomplete, thereby limiting the accuracy of ePCR simulations [41] and (2) primer specificity issues, wherein sequence variations within primer binding regions reduce primer annealing efficiency during ePCR.

This study has several limitations. First, the population genetic analyses were conducted using samples from a single location and breed (the Silver King), with a relatively limited sample size. Future studies should incorporate samples from diverse geographic regions, breeds, and feral populations to assess the generalizability. Second, although the 24 STR loci provided high discriminatory power, deviations from HWE within breeds indicate the necessity to identify and incorporate additional highly polymorphic loci that conform to HWE, thereby enhancing the reliability and efficacy of the system. Finally, cross-species analyses were limited to available high-quality reference genomes within the genus Columba, excluding many of the 34 species in the genus, and lacked empirical validation using biological samples. Future studies should aim to include a broader range of species and conduct empirical testing to further substantiate the system’s cross-species applicability.

## 4. Materials and Methods

### 4.1. Sample Collection

Blood samples were obtained from 248 unrelated individuals of the target species, the domestic pigeon (*Columba livia domestica*), procured from local farmers’ markets in Shanghai, China. All samples were collected into 2 mL EDTA anticoagulant tubes. During transportation, samples were maintained at a constant temperature of 4 °C and subsequently stored at −80 °C until further processing.

### 4.2. DNA Extraction and Quantification

Genomic DNA (gDNA) was extracted from the blood samples using the QIAamp DNA Blood Mini Kit (Qiagen, Hilden, Germany). Given that domestic pigeon erythrocytes are nucleated, the standard protocol of directly adding 200 µL of blood was ineffective. Consequently, the protocol was modified by combining 10 µL of pigeon blood with 190 µL of phosphate-buffered saline (PBS), followed by adherence to the manufacturer’s manual. Specifically, the kit’s buffer and protease were added to the homogenized blood-PBS mixture, incubated at 56 °C for 10 min, then subjected to washing and elution steps. DNA concentration was quantified using the Qubit^®^4.0 fluorometer (Thermo Fisher Scientific, Waltham, MA, USA). Extracted DNA samples were stored at −80 °C until use.

### 4.3. Locus Selection

This study initially incorporated all 12 core loci and 4 additional loci recommended by ISAG for pigeon genetic diversity and individual identification. Subsequently, eight additional loci were selected based on published literature on pigeon STRs to enhance discriminatory power [13,15,27,28]. The selection criteria prioritized loci with trinucleotide or longer repeat motifs to minimize mutation rates and ensured loci were distributed across different chromosomes to reduce linkage disequilibrium.

### 4.4. Allelic Ladder Construction

Common allele variants from 24 STR loci in domestic pigeons were used to construct an allelic ladder. For each STR locus, plasmids representing different alleles with defined repeat numbers were synthesized. For STR loci with dinucleotide core repeats, a single nucleotide insertion was introduced every six repeats within the core region, followed by plasmid synthesis corresponding to the adjusted repeat counts. The plasmid constructs underwent PCR amplification, dilution, mixing, and analysis to optimize peak height consistency and concentration. The finalized allelic ladder was validated for allele size precision. Based on the ladder data, panel and bin files were generated for use with GeneMapper ^TM^ ID-X software v1.5.

### 4.5. Multiplex PCR Amplification

Primer targeting the 24 STR loci and the sex-determining locus were redesigned and organized into six multiplex panels according to amplicon size and fluorescent labels: Set 1 (FAM-labeled): CliμD11, PIGN15, PG1, PIGN10, CliµT43, PG7; Set 2 (HEX-labeled): CliμD17, PIGN57, CliμT13, PIGN04, PIGN26; Set 3 (TAMRA-labeled): CliμD01, PG6, CliμD19, PIGN12; Set 4 (ROX-labeled): CliμT02, CliμD32, CliμT17, CHD, CliμD16; Set 5 (PUR-labeled): PG4, CliμD35, C12L4, UU-Cli11, PG5. PCR reactions were conducted in a 9 µL volume comprising 4.5 µL of 2 × CW Mix (Microread Genetics, Beijing, China), 1.8 µL of Pigeon Primer Mix (Microread Genetics, Beijing, China), 2.2 µL of nuclease-free water, and 0.5 µL of DNA template. Amplification was performed on a GeneAmp^TM^ PCR System 9700 thermal cycler (Thermo Fisher Scientific, South San Francisco, CA, USA) under the following conditions: initial denaturation at 95 °C for 5 min; 27 cycles of 95 °C for 30 s and 60 °C for 80 s; and a final extension at 60 °C for 30 min.

### 4.6. Capillary Electrophoresis and Data Analysis

PCR amplification products were separated via capillary electrophoresis. For each sample, 1 µL of the multiplex PCR amplification product was combined with 8.8 µL of HiDi Formamide and 0.2 µL of Microreader Size Standard QD550 mixture (Microread Genetics, Beijing, China). After brief centrifugation, samples were denatured at 95 °C for 3 min and immediately chilled on ice for 3 min. Electrophoresis was conducted using an ABI 3500xL Genetic Analyzer (Thermo Fisher Scientific, South San Francisco, CA, USA). Data analysis of capillary electrophoresis results was performed using GeneMapper^TM^ ID-X software v1.5 (Thermo Fisher Scientific, South San Francisco, CA, USA), with a peak height detection threshold set at 50 relative fluorescence units (RFUs). Pigeon-Control DNA (Microread Genetics, Beijing, China) served as the positive control, whereas nuclease-free water was used as the negative control.

### 4.7. Developmental Validation

The amplification and analytical procedures for subsequent experiments adhered to the protocols outlined in Section 4.5 and Section 4.6.

#### 4.7.1. Species Specificity Tests

The species specificity of the system was evaluated by testing nine commonly encountered species: human (*Homo sapiens*), monkey (*Macaca mulatta*), cow (*Bos taurus*), sheep (*Ovis aries*), horse (*Equus caballus*), pig (*Sus scrofa*), dog (*Canis lupus familiaris*), cat (*Felis catus*), mouse (*Mus musculus*), chicken (*Gallus gallus*), and duck (*Anas platyrhynchos*). Enzyme-free water was used instead of animal DNA as the negative control. For each species, three DNA samples were selected, amplified, and analyzed using the developed system, with each sample subjected to three technical replicates.

Moreover, we have conducted additional specificity tests of the panel using five avian taxa: *Spilopelia chinensis*, *Pica serica*, *Phylloscopus inornatus*, *Pycnonotus sinensis*, and *Phasianus colchicus*.

#### 4.7.2. Sensitivity Tests

To evaluate the sensitivity of the system, gradient dilution experiments were performed using DNA samples from two domestic pigeons (one female and one male) exhibiting heterozygous genotypes at most loci. The DNA was serially diluted with nuclease-free water to concentrations of 4 ng/µL, 2 ng/µL, 1 ng/µL, 0.5 ng/µL, 0.25 ng/µL, 0.125 ng/µL, and 0.0625 ng/µL. A constant volume of 0.5 µL DNA template was used for all reactions. Equal volumes of enzyme-free water were used as the negative control for the experiment. Amplification and analysis were performed as described above, with each dilution tested in triplicate. Furthermore, we performed a supplementary experiment to simulate DNA degradation. Specifically, DNA (0.5 ng/µL) extracted from two individuals was irradiated with UV light (39 W, Thermo Scientific 1300 Series A2 biological safety cabinet; Thermo Fisher Scientific, Waltham, MA, USA) at a distance of 10 cm for 0, 2, 5, 10, 20, 40, and 60 min. After irradiation, 1 µL of each sample was used for PCR amplification and electrophoresis.

#### 4.7.3. Precision Tests

Allelic ladder precision was evaluated by analyzing the allelic ladder using the ABI 3500xL Genetic Analyzer, loading standards into 24 individual capillaries. An equal volume of enzyme-free water was added as a negative control. The mean fragment size and standard deviation for each allele were calculated based on the results.

#### 4.7.4. Stability Tests

The system’s resistance to common PCR inhibitors was assessed by introducing six inhibitors (hematin, calcium ions, humic acid, indigotin, EDTA, and ethanol) into DNA samples used in the sensitivity assays (Section 2.6). 1 µL of enzyme-free water instead of the inhibitor was used as a negative control. Concentrations of inhibitors added to the reaction mixtures were based on the study by Liu [42] and are detailed in Table S12.

#### 4.7.5. Repeatability and Reproducibility Tests

Repeatability and reproducibility were evaluated by performing three independent amplifications and analyses on two randomly selected domestic pigeon DNA samples. These procedures were conducted by two different operators using the same ABI 3500xL Genetic Analyzer under identical conditions. The resulting data were used to evaluate the system’s consistency.

#### 4.7.6. Concordance Tests

Concordance of STR genotyping results was examined across various tissues (blood, liver, myocardium, stomach tissue, skeletal muscle, and saliva) obtained from a single domestic pigeon.

#### 4.7.7. Stutter Analysis

Stutter peaks, which commonly arise as PCR artifacts at STR loci and complicate genotyping, were defined as peaks differing by one repeat unit from the true allele with peak heights not exceeding 15% of the true allele peak. Using genotyping data from 248 domestic pigeon DNA samples, the minus stutter ratio (peak one repeat unit smaller than the true allele) and plus stutter ratio (peak one repeat unit larger than the true allele) were calculated for each locus. For each locus, the mean, standard deviation, and occurrence rate of minus and plus stutter were determined. Stutter ratios were calculated by dividing the stutter peak height by the true allele peak height.

#### 4.7.8. Population Genetics Analysis

A total of 248 domestic pigeon DNA samples were amplified and analyzed using the system. Population genetic parameters, including observed heterozygosity (Ho), expected heterozygosity (He), polymorphism information content (PIC), discrimination power (PD), probability of exclusion (PE), and Typical Paternity Index (TPI), were calculated using the STRAF online tool (http://www.cmpg.iee.unibe.ch/services/shiny/index_eng.html, accessed on 4 September 2025) [43]. Linkage disequilibrium (LD) testing was also performed via this platform, with *p*-values adjusted by Bonferroni correction. Genotype frequencies and allele numbers (Na) were obtained using Arlequin 3.5.2.2 [44]. Hardy–Weinberg equilibrium (HWE) was assessed with Bonferroni-corrected *p*-values. Additionally, cumulative probability of exclusion (CPE) and the combined power of discrimination (CPD) were calculated as follows [45]:

(1) $$CPE=1-\prod_{i=1}^{K}\left(1-P_{i}\right)$$

(2) $$TDP=1-\prod_{k=1}^{K}\left(1-P_{k}\right)$$

where *P_i_* denotes the *i*-th PE value, *P_k_* indicates the *k*-th PD value, and *K* is the total number of PE or PD values.

#### 4.7.9. Data Analysis and Quality Control

Population genetics data were analyzed using the software described in Section 4.7.8. Other data analysis and visualizations were conducted using R 4.3.3 and GraphPad Prism 10.4.1. All methodologies conformed to the guidelines recommended by the International Society for Forensic Genetics (ISFG) [20] and the Scientific Working Group on DNA Analysis Methods (SWGDAM) [46].

#### 4.7.10. Cross-Species Applicability

To evaluate the applicability of the system to other species within the genus Columba, electronic PCR was performed using the primers designed for domestic pigeon loci via the NCBI Primer-BLAST tool (https://www.ncbi.nlm.nih.gov/tools/primer-blast/, 20 May 2025). Amplified sequences were retrieved for comparative analysis. Reference genome sequences for four Columba species, including *Columba livia domestica* (GCF_036024875.1), *Columba guinea* (GCA_032206185.1), *Columba janthina* (GCA_014362705.1), and *Columba palumbus* (GCA_965250375.1), were downloaded from the NCBI database. A local reference genome database was constructed using locus sequences from these four species. Each domestic pigeon reference sequence was aligned against the four reference genomes using local BLASTn 2.16.0 analysis to identify homologous sequences. Amplified sequences, including 500 bp upstream and downstream, were extracted for further analysis. Multiple sequence alignments were performed using MEGAX software 10.2.6 employing the MUSCLE algorithm. Genetic distances between the domestic pigeon and the other three species were calculated using p-distance metrics. Additionally, base repeat motifs and repeat counts within each homologous sequence were characterized.

## Figures and Tables

**Table 1:** Frequencies of alleles in the 24 microsatellite markers of domestic pigeons (n = 248).

**CliµD11**		**PIGN15**		**PG1**		**PIGN10**		**CliµT43**	
**Allele**	**Frequence**	**Allele**	**Frequence**	**Allele**	**Frequence**	**Allele**	**Frequence**	**Allele**	**Frequence**
11	0.030242	7	0.096774	15	0.004032	10	0.21169	10	0.048387
12	0.060484	8	0.17137	16	0.008065	11	0.125	11	0.14919
13	0.43548	9	0.030242	17	0.004032	12	0.004032	12	0.03629
14	0.002016	10	0.16935	18	0.35887	13	0.016129	13	0.004032
15	0.13105	11	0.21371	19	0.41532	13.1	0.002016	14	0.03629
18	0.090726	12	0.20968	20	0.17742	13.2	0.034274	15	0.22379
20	0.17944	13	0.10887	21	0.02621	14	0.034274	16	0.35282
21	0.070565	**CliµD17**		22	0.006048	15	0.08871	17	0.14315
**PG7**		**Allele**	**Frequence**	**PIGN57**		15.2	0.002016	18	0.006048
**Allele**	**Frequence**	10	0.010081	**Allele**	**Frequence**	16	0.15726	**PIGN04**	
11	0.3125	11	0.125	7	0.032258	17	0.070565	**Allele**	**Frequence**
19.1	0.32661	12	0.092742	8	0.24597	17.2	0.050403	10	0.030242
21.2	0.10484	13	0.010081	9	0.004032	18	0.002016	11	0.32056
22.1	0.006048	14	0.006048	11	0.12097	18.2	0.072581	12	0.012097
22.2	0.002016	15	0.1875	12	0.35081	19.2	0.018145	13	0.18347
23.1	0.2379	16	0.54234	13	0.10685	20.2	0.074597	14	0.074597
24.1	0.010081	17	0.006048	14	0.12298	21	0.03629	15	0.014113
**PIGN26**		18	0.020161	15	0.010081	**CliµT13**		16	0.054435
**Allele**	**Frequence**	**CliµD01**		16	0.006048	**Allele**	**Frequence**	17	0.10887
9	0.014113	**Allele**	**Frequence**	**PG6**		8	0.032258	18	0.16129
11	0.038306	8	0.002016	**Allele**	**Frequence**	9	0.002016	19	0.03629
14	0.024194	13	0.37903	5	0.14113	12	0.002016	21	0.002016
15	0.014113	14	0.006048	6	0.024194	13	0.066532	22	0.002016
15.1	0.018145	15	0.018145	7	0.74194	14	0.048387	**PIGN12**	
16	0.032258	16	0.002016	8	0.092742	15	0.15323	**Allele**	**Frequence**
16.1	0.012097	17	0.006048	**CliµT17**		16	0.31653	15.4	0.002016
17	0.022177	18	0.11492	**Allele**	**Frequence**	17	0.18952	16.2	0.002016
17.1	0.014113	19	0.10484	5	0.32056	18	0.15121	17	0.02621
18	0.15121	20	0.0625	7	0.14315	19	0.03629	17.4	0.004032
18.1	0.024194	21	0.016129	8	0.125	20	0.002016	18	0.014113
19	0.0625	23	0.040323	9	0.002016	**CliµD19**		19	0.012097
19.1	0.008065	24	0.002016	10	0.034274	**Allele**	**Frequence**	20	0.03629
20	0.050403	26	0.056452	11	0.006048	11	0.016129	21	0.11694
21	0.16331	27	0.056452	12	0.046371	12	0.002016	22	0.15927
22	0.096774	28	0.12702	13	0.20766	13	0.020161	22.4	0.002016
23	0.10685	29	0.004032	14	0.11492	14	0.51815	23	0.20565
24	0.048387	30	0.002016	**CliµD16**		15	0.010081	23.4	0.030242
25	0.024194	**CliµD32**		**Allele**	**Frequence**	16	0.43347	24	0.030242
26	0.022177	**Allele**	**Frequence**	10	0.090726	**C12L4**		24.4	0.002016
27	0.02621	5.1	0.080645	11	0.006048	**Allele**	**Frequence**	25	0.010081
28	0.012097	6	0.004032	12	0.10887	10	0.004032	26	0.022177
28.1	0.002016	6.1	0.006048	14	0.59073	11.1	0.012097	27	0.002016
29	0.004032	7	0.012097	16	0.012097	12	0.40121	29	0.020161
30	0.002016	8	0.012097	17	0.02621	12.1	0.004032	29.3	0.004032
31	0.002016	8.1	0.032258	18	0.018145	13	0.020161	30.2	0.002016
32	0.004032	9	0.1875	22	0.064516	13.1	0.006048	30.3	0.006048
**PG4**		10	0.002016	25	0.024194	14	0.006048	31	0.004032
**Allele**	**Frequence**	11	0.15726	26	0.002016	15	0.050403	31.3	0.008065
11	0.15524	12	0.14516	28	0.004032	15.1	0.018145	32	0.002016
12	0.054435	13	0.28427	29	0.012097	16	0.056452	32.2	0.012097
14	0.008065	14	0.004032	30	0.004032	17	0.042339	32.3	0.02621
15	0.13508	16	0.056452	32	0.034274	18	0.26411	33	0.010081
16	0.36492	17	0.006048	33	0.002016	19	0.008065	33.2	0.082661
17	0.27419	18	0.010081	**UU-Cli11**		20	0.10685	33.3	0.070565
18	0.008065	**CliµT02**		**Allele**	**Frequence**	**PG5**		34	0.038306
**CliµD35**		**Allele**	**Frequence**	9.1	0.16129	**Allele**	**Frequence**	34.2	0.024194
**Allele**	**Frequence**	11	0.006048	9.2	0.042339	10.3	0.81048	34.3	0.002016
5.1	0.002016	12	0.1875	10.1	0.77016	11.3	0.18952	35	0.006048
11	0.042339	13	0.53831	10.2	0.014113			35.2	0.004032
12	0.84274	14	0.23387	11	0.002016				
13	0.074597	15	0.008065	11.2	0.010081				
14	0.034274	16	0.004032						
15	0.004032	17	0.022177						

## Data Availability

The original contributions presented in this study are included in the article and Supplementary Materials. Further inquiries can be directed to the corresponding authors.

## Supplementary Materials

The following supporting information can be downloaded at https://www.mdpi.com/article/10.3390/ijms27188324/s1.
