# Supplementary material for: Development and Validation of a Novel Forensic STR Multiplex Assay for Domestic Pigeon (Columba livia domestica)
Source: Int J Mol Sci. 2026 Sep 19;27(18):8324. doi: 10.3390/ijms27188324 (PMC13607457; doi:10.3390/ijms27188324)
Supplement: Supplementary file 1 [file ijms-27-08324-s001.zip › Figure legends.pdf]

Figure legends

Figure S1. Distribution of repeat units across 24 pigeon gene locations. 2bp: dinucleotide repeat motif; 3bp: trinucleotide repeat motif; 4bp: tetranucleotide repeat motif; 5bp: pentanucleotide repeat motif; com: compound STR motif. The x-axis represents the number of repeat bases, and the y-axis represents the number of loci.

Figure S2. Electropherogram of the allelic ladder constructed for the system.

Figure S3. Results of DNA amplification analysis performed on the pigeon sample designated CL28 using this system. The instrument employed was the ABI 3500xL Genetic Analyzer.

Figure S4. Species-specific testing in 11 species. Species include human (*Homo sapiens*), monkey (*Macaca mulatta*), cow (*Bos taurus*), sheep (*Ovis aries*), horse (*Equus caballus*), pig (*Sus scrofa*), dog (*Canis lupus familiaris*), cat (*Felis catus*), mouse (*Mus musculus*), chicken (*Gallus gallus*) and duck (*Anas platyrhynchos*). Three replicates per species.

Figure S5. Species-specific testing in five avian species. Species include *Spilopelia chinensis, Pica serica, Phylloscopus inornatus*, *Pycnonotus sinensis*, and *Phasianus colchicus.* Three replicates per species.
