# Supplementary material for: Development and Validation of a Novel Forensic STR Multiplex Assay for Domestic Pigeon (Columba livia domestica)
Source: Int J Mol Sci. 2026 Sep 19;27(18):8324. doi: 10.3390/ijms27188324 (PMC13607457; doi:10.3390/ijms27188324)
Supplement: Supplementary file 1 [file ijms-27-08324-s001.zip › Figure S1.pdf]

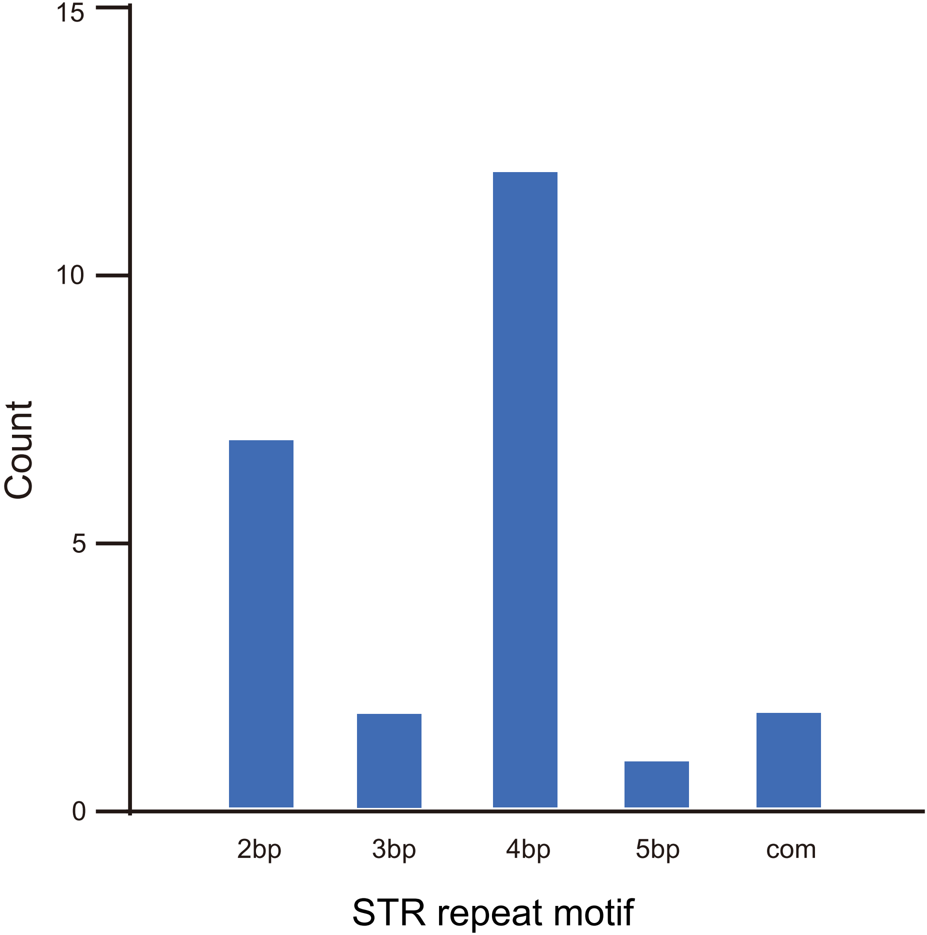
15
10
5
0
Count
2bp
3bp
4bp
5bp
com
STR repeat motif
