# Supplementary material for: Development and Validation of a Novel Forensic STR Multiplex Assay for Domestic Pigeon (Columba livia domestica)
Source: Int J Mol Sci. 2026 Sep 19;27(18):8324. doi: 10.3390/ijms27188324 (PMC13607457; doi:10.3390/ijms27188324)
Supplement: Supplementary file 1 [file ijms-27-08324-s001.zip › Figure S2.pdf]

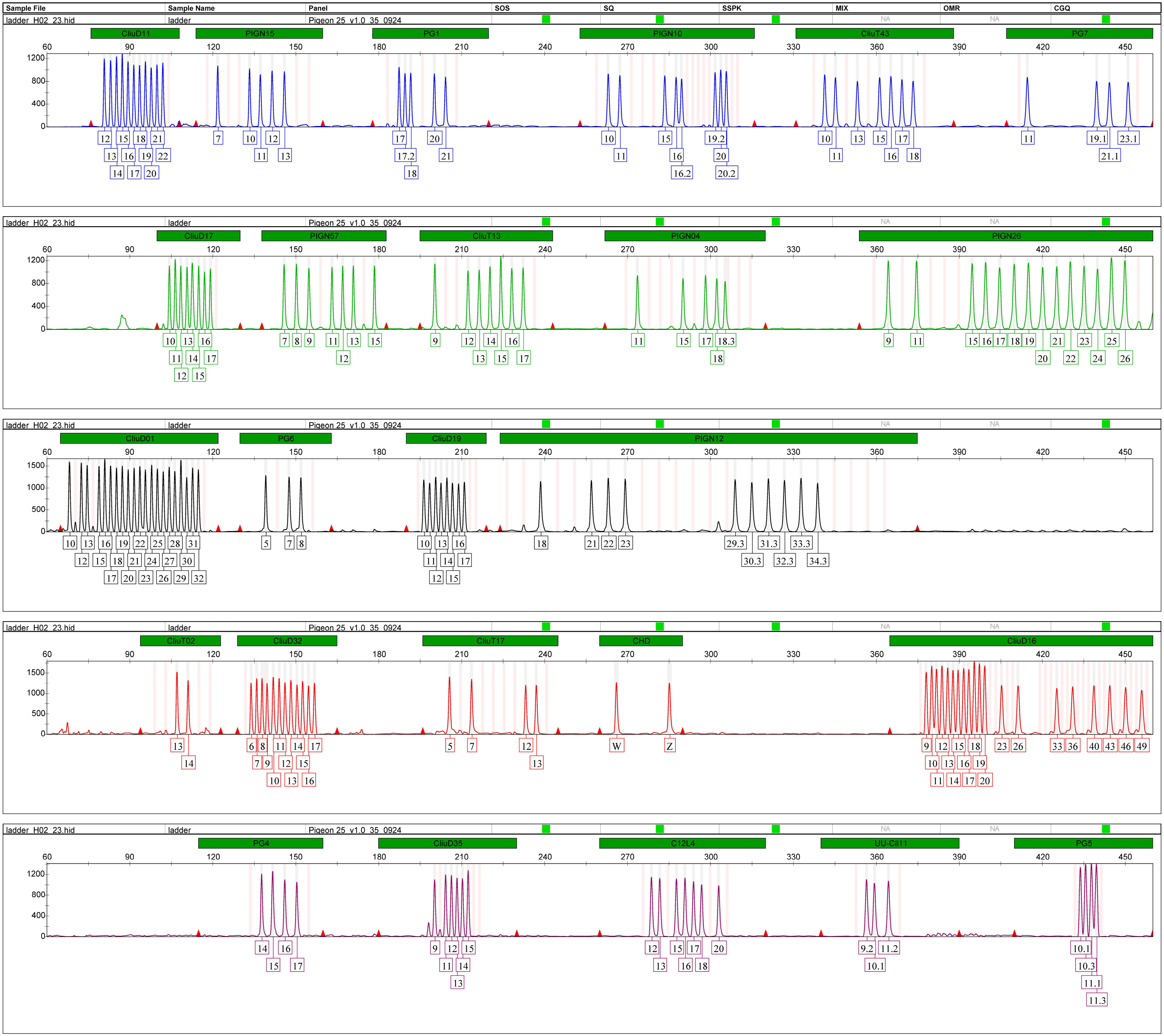

Sample File
Sample Name
Panel
SOS
SQ
SSPK
MIX
OMR
CGQ
ladder_H02_23.hid
ladder
Pigeon 25_v1.0_35_0924
NA
NA
CliuD11
PIGN15
PG1
PIGN10
CliuT43
PG7
12 13 14 15 16 17 18 19 20 21 22
7
10 11 12 13
17 17.2 18 20 21
10 11
15 16 16.2 19.2 20 20.2
10 11 13 15 16 17 18
11
19.1 21.1 23.1
CliuD17
PIGN57
CliuT13
PIGN04
PIGN26
10 11 12 13 14 15 16 17
7 8 9 11 12 13 15
9 12 13 14 15 16 17
11 15 17 18 18.3
9 11 15 16 17 18 19 20 21 22 23 24 25 26
CliuD01
PG6
CliuD19
PIGN12
10 12 13 15 16 17 18 19 20 21 22 23 24 25 26 27 28 29 30 31 32
5 7 8
10 11 12 13 14 15 16 17
18 21 22 23
29.3 30.3 31.3 32.3 33.3 34.3
CliuT02
CliuD32
CliuT17
CHD
CliuD16
13 14
6 7 8 9 10 11 12 13 14 15 16 17
5 7 12 13
W Z
9 10 11 12 13 14 15 16 17 18 19 20 23 26 33 36 40 43 46 49
PG4
CliuD35
C12L4
UU-Cli11
PG5
14 15 16 17
9 11 12 13 14 15
12 13 15 16 17 18 20
9.2 10.1 11.2
10.1 10.3 11.1 11.3
