# Supplementary material for: Development and Validation of a Novel Forensic STR Multiplex Assay for Domestic Pigeon (Columba livia domestica)
Source: Int J Mol Sci. 2026 Sep 19;27(18):8324. doi: 10.3390/ijms27188324 (PMC13607457; doi:10.3390/ijms27188324)

Sample File	Sample Name	Panel	SOS	SQ	SSPK	MIX	OMR	CGQ
CL28_C10_07.hid	CL28	Pigeon_25_v1.0_35_0924						

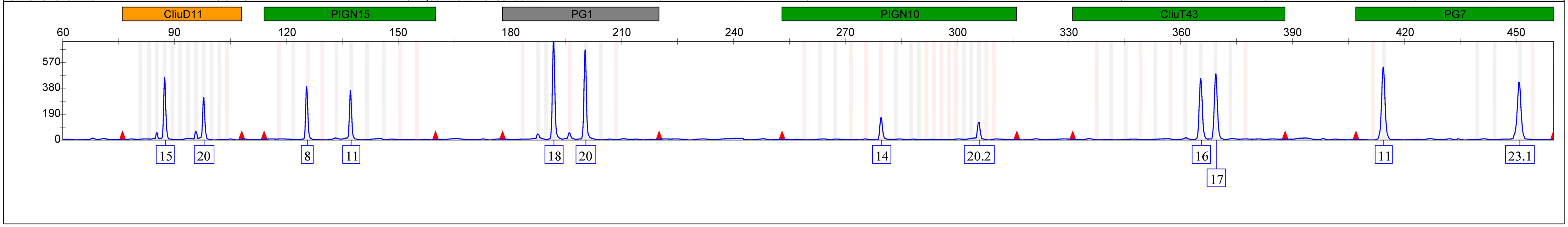

CL28_C10_07.hid | CL28 | Pigeon_25_v1.0_35_0924

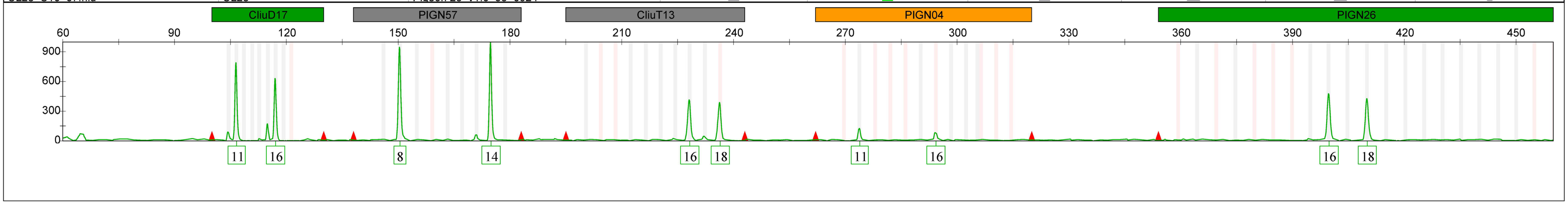

CL28_C10_07.hid | CL28 | Pigeon_25_v1.0_35_0924

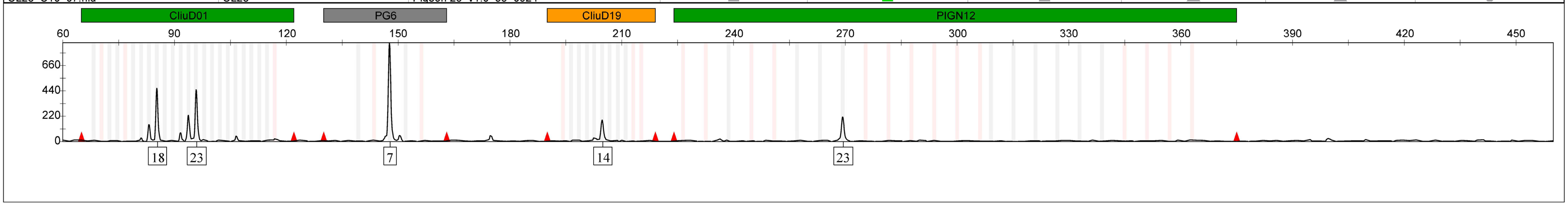

CL28_C10_07.hid | CL28 | Pigeon_25_v1.0_35_0924

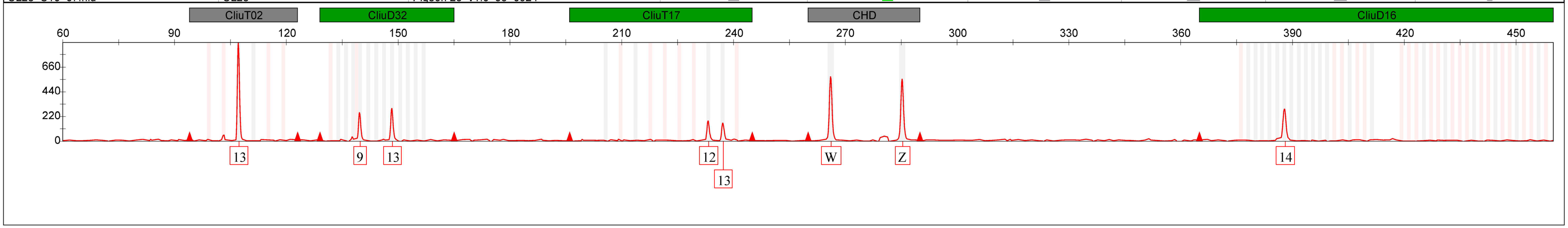

CL28_C10_07.hid | CL28 | Pigeon_25_v1.0_35_0924

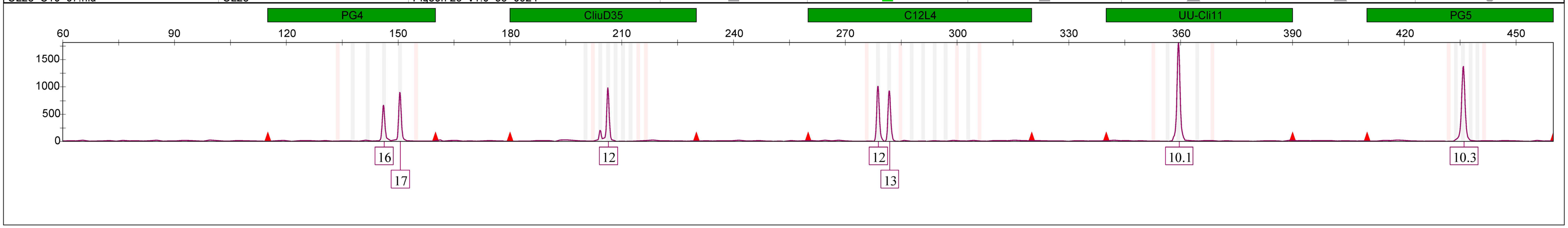

Supplement: Supplementary file 1 [file ijms-27-08324-s001.zip › Figure S3.pdf]
